# Effects of the feeding protocol during blood transfusion on splanchnic tissue oxygenation and complications in very premature infants

**DOI:** 10.3389/fnut.2024.1408717

**Published:** 2024-07-09

**Authors:** Jianghua He, Xueshi Sun, Xiaoming Xu, Hanwen Luo, Jun Tang, Tao Xiong, Jing Zhao, Jing Shi

**Affiliations:** ^1^Department of Pediatrics, West China Second University Hospital, Sichuan University, Chengdu, Sichuan, China; ^2^Key Laboratory of Obstetrics and Gynecologic and Pediatric Diseases and Birth Defects of the Ministry of Education, Sichuan University, Chengdu, Sichuan, China

**Keywords:** blood transfusion, complications, near-infrared spectroscopy, splanchnic tissue oxygenation, very premature infants

## Abstract

**Background:**

The effects of blood transfusions on splanchnic oxygenation and complications related to blood transfusions, including red blood cell (RBC) transfusions, in premature infants undergoing enteral feeding, to provide clinical evidence for a management protocol for premature infants during the peri-transfusion period.

**Methods:**

This single-blind, randomized, controlled trial enrolled sixty eligible preterm infants who were randomly divided into the withholding feeding group (*n* = 30) or feeding group (*n* = 30). Enteral feeding was withheld for 8 h, beginning from the start of transfusion infants in the feeding group were fed according to the pre-transfusion feeding approach during and after RBC transfusion.

**Results:**

Baseline characteristics of those in the withholding and feeding groups were as follows: gestational age (weeks) 27.52 (24.86–30.14) and 27.13 (25.43–30.14); birth weight (g), 1,027 (620–1,450) and 1,027 (620–1,270); blood transfusion day, 48 (14–79) and 39 (10–78); and hemoglobin before blood transfusion (g/L), 81.67 (±10.56) and 85.93 (±14.77). No significant differences were observed between groups at baseline. No significant differences were observed in the average splanchnic tissue oxygenation changes or clinical results at any time. One patient in the withholding feeding group experienced transfusion-associated necrotizing enterocolitis.

**Conclusions:**

No differences in splanchnic oxygenation observed these feeding protocols. This study suggests the feasibility of a sizable trial to evaluate clinical outcomes. The risks of mesenteric ischemia and transfusion-related necrotizing enterocolitis for premature infants were not increased by enteral feeding during RBC transfusion.

**Clinical trial registration:**

ChiCTR2200055726 (https://www.chictr.org.cn/).

## Background

Premature infants are at high risk for anemia development because of their unique physiological conditions and iatrogenic causes. During the initial stay in the neonatal intensive care unit (NICU), >60% of very preterm infants and 90% of infants with extremely low birth weight receive blood transfusions (1). Blood transfusions can lead to specific complications, such as transfusion-associated necrotizing enterocolitis (TA-NEC) (2). Additionally, blood transfusions for preterm infants may increase the risks of retinopathy of prematurity (ROP), intraventricular hemorrhage (IVH), and bronchopulmonary dysplasia (BPD) (3).

TA-NEC refers to NEC episodes that are temporally related to packed red blood cell (RBC) transfusion and typically occur within 48 h after transfusion (4, 5). According to several studies (6, 7), 25%−35% of infants with NEC received blood transfusions prior to being diagnosed. Packed RBC transfusions, enteral feedings, and gastrointestinal immaturity are all risk factors for TA-NEC (8). Anemia can lead to intestinal ischemia and hypoxia, and blood transfusions can lead to intestinal tissue reperfusion and oxidative stress damage, thus increasing the risk of TA-NEC (9). Using near-infrared spectroscopy (NIRS) to monitor the blood oxygen saturation of local intestinal tissues, one study showed that the blood oxygen saturation of the mesentery in infants with TA-NEC decreased more significantly than that in infants without NEC after blood transfusion (10). Infants, particularly premature infants, have immature autoregulation of intestinal blood flow. If the intestine cannot obtain sufficient oxygen, then the local tissues may experience hypoxia and ischemia, leading to TA-NEC. Feeding during blood transfusions for preterm infants with anemia leads to decreased intestinal oxygenation, which may be related to TA-NEC (11, 12).

Withholding enteral feeding during blood transfusions for preterm infants may prevent TA-NEC. One systematic review of non-randomized controlled trials (non-RCTs) that included seven studies and 7,492 infants found that when feeding was withheld throughout the transfusion, the odds ratio (OR) of NEC development within 48–72 h was significantly low [OR, 0.47; 95% confidence interval (CI), 0.28–0.80] (13). A recent Cochrane review (14) (including one RCT and 22 preterm infants) found evidence that could not sufficiently suggest that delaying enteral feeding for infants who needed blood transfusions had an impact on NEC development. An RCT with a small sample size that divided preterm infants into three groups according to the feeding protocol found no significant differences in the mean splanchnic cerebral oxygenation ratio (SCOR), mean splanchnic fractional oxygen extraction (FOE), and TA-NEC when enteral feeds were withheld, continued, and restricted during blood transfusions; however, the feeding intolerance (FI) incidences were 0%, 25%, and 5% (*P* = 0.02), respectively (15).

Studies of feeding and NEC during blood transfusions for premature infants have primarily focused on preterm infants and a feeding amount ≥120 ml/kg. However, clinically, many premature infants receive blood transfusions before their feeding amount reaches 120 ml/kg. Moreover, studies have not limited the frequency of blood transfusions for the included infants. Multiple blood transfusions are associated with the severity of intestinal mucosal damage (16). One case study revealed that the administration of two full-volume transfusions within a relatively short timeframe was associated with pneumoperitoneum and TA-NEC (17). Therefore, this study aimed to examine the changes in splanchnic tissue oxygenation during the implementation of a protocol comprising the withholding of enteral feeding compared with those associated with routine enteral feeding during the peri-transfusion period for preterm infants who received partial enteral nutrition. Furthermore, this study analyzed the effects of blood transfusion and various feeding strategies on splanchnic tissue oxygenation and transfusion-related complications among very premature infants to provide clinical evidence for a management protocol for premature infants who require RBC transfusions.

## Methods

### Study design and participants

This single-center, single-blind RCT investigated the effects of different enteral feeding strategies on splanchnic oxygenation and transfusion-related complications during RBC transfusion. This study followed the Consolidated Standards of Reporting Trials (CONSORT) reporting guidelines. The participants were preterm infants admitted to the NICU at the West China Second University Hospital of Sichuan University who required blood transfusions because of anemia. They were enrolled between February 2022 and January 2023. The West China Second University Hospital of Sichuan University is a tertiary hospital for women and children. The neonatal division is one of the largest neonatal wards in China, with nearly 6,000 infants discharged annually, including 400 very premature infants.

The eligibility criteria for study inclusion were as follows: preterm infants born at <32 weeks of gestation; received one or more RBC transfusions for anemia; birth weight <1,500 g; and enteral feeding amount ≥60 ml/kg/day. All RBC transfusions performed during this study were administered following the guidelines of the British Committee for Standards in Hematology (18).

The exclusion criteria were as follows: major chromosomal disorders or congenital abnormalities; IVH with a Papile grade of III or higher; hemodynamically significant patent ductus arteriosus; severe infection; NEC; active bleeding; small for gestational age status; vasopressor therapy required at the study entry point; and cutaneous disease that did not allow the placement of an NIRS sensor. The trial was approved by the institution's Ethics Committee (no. 064, Medical Research 2021 Ethical Review, June 25, 2021) and registered at chictr.org (ChiCTR2200055726). Written informed consent was obtained from the parents of the participants before recruitment.

### Interventions

Infants who met the inclusion criteria were randomly divided into the feeding and withholding feeding groups. Enteral feeds were stopped for 8 h, from the start of the transfusion, for infants in the withholding feeding group. Infants in the feeding group received orogastric tube feeding via gravity for 5–10 min every 3 h before transfusion according to the protocol. During the transfusion, infants who did not receive enteral feeds were administered intravenous fluids to maintain their blood sugar levels. All transfusions were administered 4 h and comprised leukocyte-reduced RBCs (20 cc/kg) from the local blood bank. The blood was irradiated immediately before transfusion. Blood products were heated using a transfusion heater to maintain a temperature of 37°C. The ventilator's settings and inhaled oxygen concentration for infants who needed respiratory support and oxygen inhalation were adjusted to keep their oxygen levels between 90 and 94%. Each transfusion episode was treated as a separate event. Infants were assessed for eligibility and randomized with each transfusion.

### NIRS measurements

Using the NIRS parameter monitor/brain oxygen monitor (EGOS-600B; Aiqin Medical Biomedical Electronics, Suzhou, China), the regional oxygen saturation (rSO_2_) of each patient was assessed at predetermined time points. Neonatal sensors were secured with tape at the correct location 1 h before the transfusion and removed 48 h after the transfusion. The skin integrity of each infant was checked every 6 h by the study nurses. To evaluate cerebral, hepatic, renal, and intestinal splanchnic tissue oxygenation, neonatal sensors were taped to the lateral sides of the forehead (19), on the right flank (last rib level) (20), left posterolateral flank (21), and left lower abdominal quadrant (19).

According to the operating manual of the NIRS monitor and previous studies (22, 23), a period of 10 min was allotted for each measurement; thereafter, the NIRS stability curve value was recorded, and the average of the measurement data was obtained. Clinical symptoms were observed and recorded, and the SPO_2_, blood pressure, and heart rate were simultaneously monitored and recorded.

The SCOR and cerebral and splanchnic FOE were determined based on the average raw data of regional splanchnic saturation measured using NIRS. The following calculations were applied (24):


$$\begin{array}{l} \text{SCOR = intestinal }{\text{r}}_{\text{SO2}}\text{/cerebral }{\text{r}}_{\text{SO2}}\\ \text{ FOE = }\left(\left[{\text{SpO}}_{\text{2}}\;{\text{rSO}}_{\text{2}}\right]\times \frac{\text{100}}{\left[{\text{SpO}}_{\text{2}}\right]}\right). \end{array}$$

### Primary outcomes

The primary outcomes were the mean rSO_2_ measurements during the following five periods: before the transfusion (30 min before transfusion); at the end of the transfusion; and after the transfusion (8, 12, and 24 h after transfusion). The mean SCOR and splanchnic FOE measurements were also primary outcomes.

### Secondary outcomes

The secondary outcomes were gastrointestinal complications within 48 h after transfusion, FI, TA-NEC, and the incidence of complications among premature infants during hospitalization, including ROP, BPD, NEC, and IVH. FI was defined as gastric aspirates >50% of feed volume, vomiting, or failure of the feeding protocol, including the reduction, postponement, or cessation of enteral nutrition (25). TA-NEC was defined as a diagnosis of NEC within 48 h after transfusion (26).

### Sample size

Based on studies of anemia in very preterm infants, we estimated that the mean intestinal oxygen saturations would be between 40 and 50%, with a standard variation of 7% (27, 28). To achieve 90% power at 0.05, it was estimated that a sample size comprising ≥12 infants was required to detect a 15% change in mean splanchnic oxygenation. Considering the risk of patient loss, we recruited 30 infants for each group.

### Randomization and blinding

Infants were randomized using a random number table and 1:1 ratio. This was a single-blind study for NIRS measurement staff, feeding regimen assignments were not blinded to other clinical staff members.

### Statistical analysis

Data analyses were performed using the SPSS 25.0 statistical program. Means and standard deviations were used to describe normally distributed data. Means (minimum, maximum) were used to represent non-normally distributed data. Counting data were described as numbers (%). To compare the means of independent sample data, a *t*-test was performed. For cases with data that were not normally distributed, a rank sum test was performed. Enumeration data were analyzed using the squared test. analysis of variance of repeated measurements of two factors was performed to assess the local oxygenation values of splanchnic tissue oxygenation in the two groups of children. These values were measured repeatedly at different time points. Statistical significance was set at *P* < 0.05.

## Results

**Figure 1:**
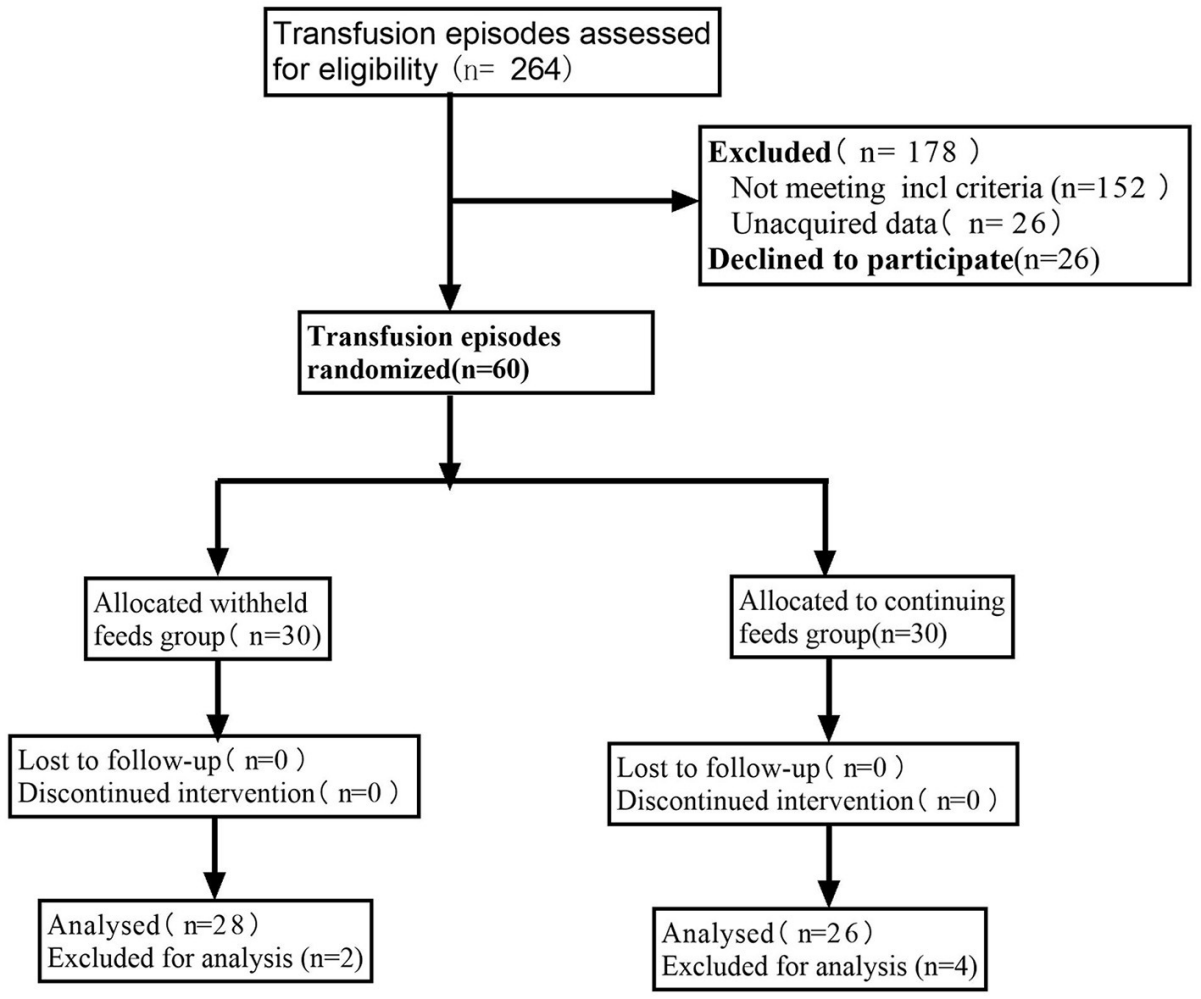
Consolidated standards of reporting trials (CONSORT) diagram of the study participant flow.

Figure 1 presents the CONSORT recruiting diagram. Sixty infants met the inclusion criteria; therefore, 30 infants were assigned to each group. All parents or legal guardians of the included infants provided informed consent. Because of inadequate data, the study excluded two and four events from the feeding and withholding feeding groups, respectively.

Table 1 shows the demographic and clinical characteristics of each group at baseline. Differences in baseline characteristics of the infants in the withholding feeding and feeding groups were not significant. Similar prenatal characteristics were observed in both groups, and effective blood transfusions were performed for each patient.

**Table 1:** Baseline characteristics of the study subjects.

	**Withholding feeding group (*N* = 28)**	**Feeding group (*N* = 26)**	***P*-value**
Gestation age at birth, weeks (minimum, maximum)	27.52 (24.86, 30.14)	27.13 (25.43, 30.14)	0.479
Male sex, *n* (%)	20 (71.4)	13 (50)	0.107
Birth weight, mean (minimum, maximum), g	1,027 (620, 1,450)	1,027 (620, 1,270)	0.256
Vaginal delivery, *n* (%)	22 (78.6)	18 (69.2)	0.434
Corrected gestational age, mean ± SD	34.14 ± 2.57	33.20 ± 3.193	0.364
Postnatal age (minimum, maximum)	48 (14, 79)	39 (10, 78)	0.57
Weight at the time of the study, mean ± SD	1.71 ± 0.41	1.55 ± 0.40	0.296
Hematocrit level before transfusion, mean ± SD	24.54 ± 3.315	25.64 ± 4.59	0.441
Hemoglobin level before transfusion, mean ± SD	81.67 ± 10.56	85.93 ± 14.77	0.349
PDA, *n* (%)	11 (39.3)	15 (57.7)	0.176
Grade I/II intraventricular hemorrhage, *n* (%)	5 (17.1)	3 (11.5)	0.209
**Feeding type at the time of the study**			0.504
Breastfeeding, *n* (%)	8 (28.6)	4 (15.4)	
Milk feeding *n* (%)	3 (10.7)	3 (11.5)	
Mixed feeding, *n* (%)	17 (60.7)	19 (73.1)	
**Oxygen required at the time of the study**			0.905
Nasal tube oxygen at the time of the study, *n* (%)	9 (32.1)	6 (23.1)	
Non-invasive respiratory support at the time of the study, *n* (%)	14 (50)	15 (57.7)	
Invasive ventilation at the time of the study, *n* (%)	2 (7.1)	2 (7.7)	
Caffeine, *n* (%)	19 (67.9)	17 (65.4)	0.847
Dexamethasone, *n* (%)	2 (7.1)	4 (15.4)	0.336
Iron, *n* (%)	9 (32.1)	5 (19.2)	0.279
Probiotics, *n* (%)	1 (3.6)	0 (0)	0.311
Serum creatinine, mean (minimum, maximum), μmol/L	36 (2.6, 81)	35 (3.2, 64)	0.968
Blood urea nitrogen, mean (minimum, maximum), mmol/L	2.6 (1.4, 14.9)	4.7 (2, 6.6)	0.248

PDA, patent ductus arteriosus; SD, standard deviation.

In this study, transfusions were associated with an upward trend in splanchnic tissue oxygenation in both groups (Table 2). A comparison of values observed before and after transfusions showed that the feeding group a greater increase in the intestinal rSO_2_ (*T* = 2.381; *P* = 0.031), whereas the withholding feeding group experienced a greater increase in the renal rSO_2_ (*T* = 5.019; *P* < 0.001). This difference was significant.

**Table 2:** Changes in splanchnic tissue oxygenation before and at the end of blood transfusion.

	**Mean ±SD (%)**	***T***	***P*-value**
**Cerebral rSO**_2_ **T1–T0**			
Withholding feeding group	3.17 ± 7.09	1.845	0.084
Feeding group	1.61 ± 4.37	1.276	0.228
**Intestinal rSO**_2_ **T1–T0**			
Withholding feeding group	2.01 ± 8.97	0.950	0.355
Feeding group	4.44 ± 6.97	2.381	0.033
**Renal rSO**_2_ **T1–T0**			
Withholding feeding group	4.29 ± 3.42	5.019	0.001
Feeding group	−0.42 ± 8.67	−0.176	0.863
**Hepatic rSO**_2_ **T1–T0**			
Withholding feeding group	1.78 ± 8.24	0.915	0.373
Feeding group	0.55 ± 5.39	0.379	0.711

rSO_2_ T1–T0 represents the splanchnic oxygenation value at the end of the blood transfusion minus that before the transfusion.

rSO_2_, regional oxygen saturation; SD, standard deviation; T0, timepoint before transfusion; T1, timepoint after transfusion.

**Figure 2:**
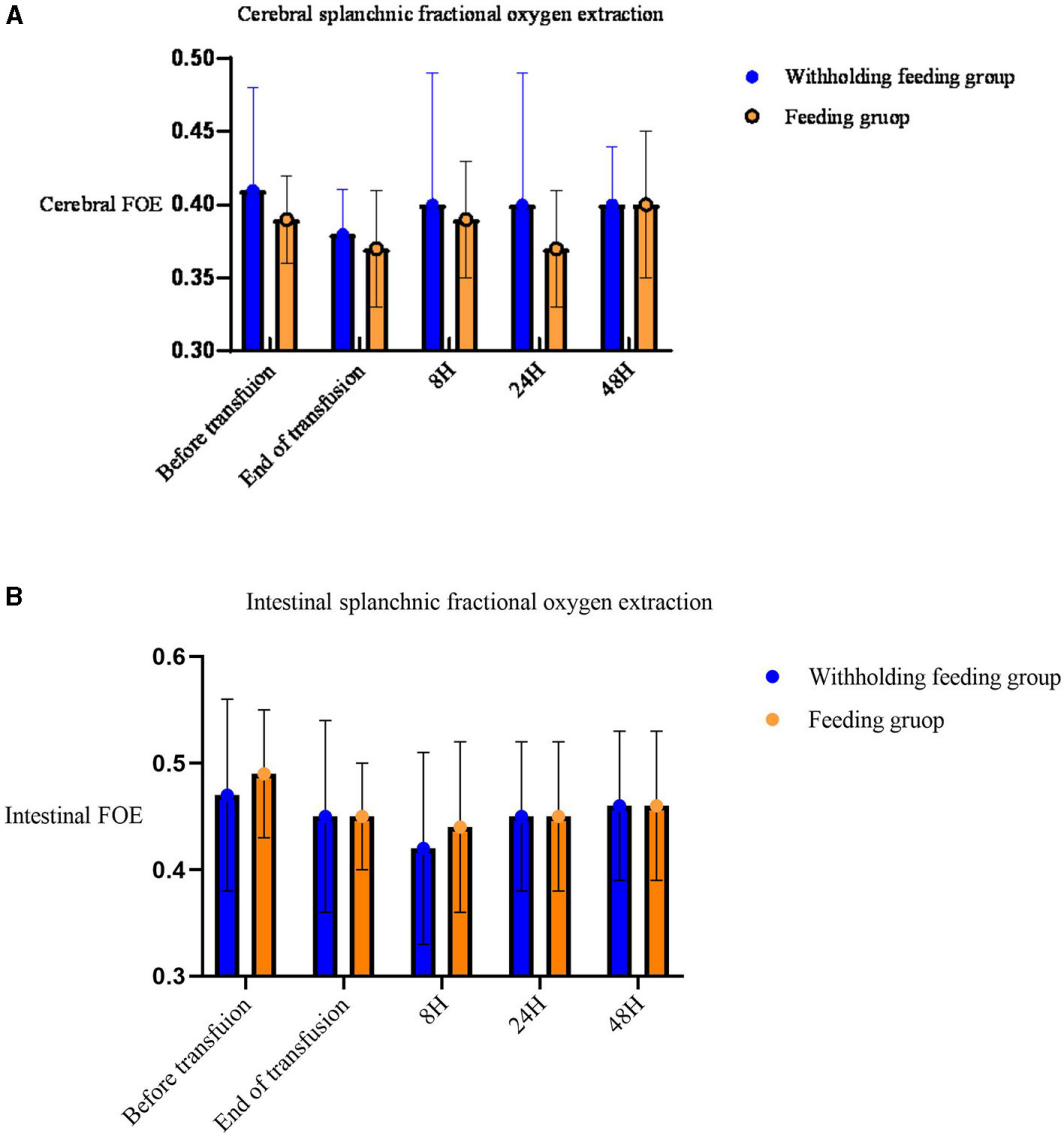
Mean (standard deviation) splanchnic fractional oxygen extraction (FOE) [(SpO_2_c- rSO_2_S)/SpO_2_]. **(A)** Cerebral FOE. **(B)** Intestinal FOE.

**Figure 3:**
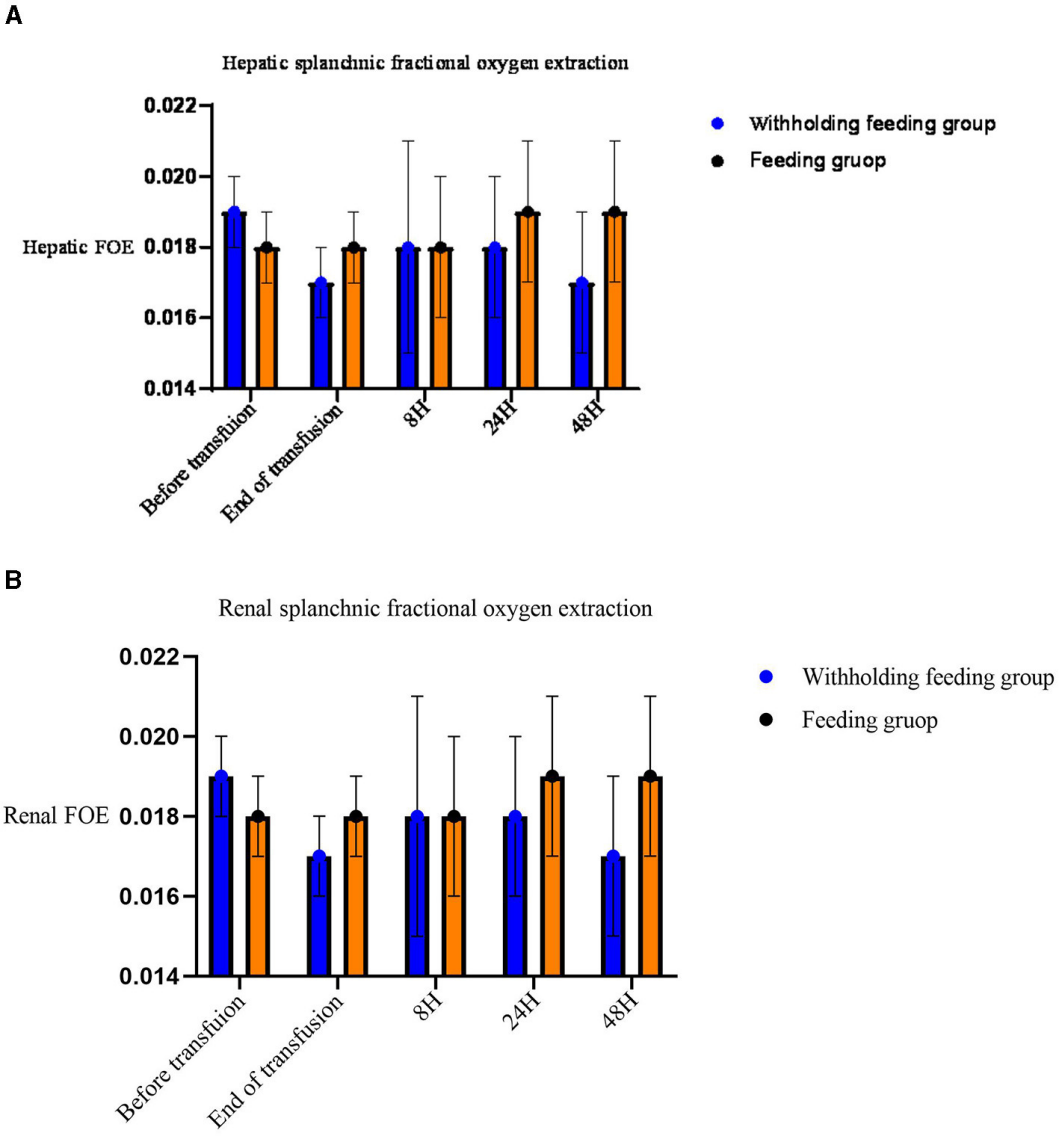
Mean (standard deviation) splanchnic fractional oxygen extraction (FOE) [(SpO_2_- crSO_2_S)/SpO_2_]. **(A)** Hepatic FOE. **(B)** Renal FOE.

No significant differences in the rSO_2_ and FOE (Table 3, Figures 2, 3) were observed in the brain, intestines, liver, and SCOR of the groups at various times before and after transfusions. No interactions between the various time points before and after transfusions and feeding strategies were observed.

**Table 3:** NIRS values of splanchnic oxygenation at prespecified time points.

**Time point**	**Before transfusion**	**End of transfusion**	**8 h**	**24 h**	**48 h**
**Cerebral rSO**_2_					
Withholding feeding group	55.56 (6.03)	58.68 (2.6)	57.09 (8.52)	56.31 (8.31)	56.99 (3.13)
Feeding group	57.36 (3.49)	59.19 (3.52)	57.51 (3.81)	58.99 (3.77)	56.67 (3.69)
*P*-value	0.540	0.830	0.669	0.188	0.937
**Intestinal rSO**_2_					
Withholding feeding group	50.06 (8.52)	52.07 (7.65)	54.62 (8.16)	51.97 (6.47)	51.09 (6.9)
Feeding group	47.86 (5.42)	52.06 (4.86)	52.21 (7.01)	51.8 (6.67)	50.85 (6.36)
*P*-value	0.421	0.997	0.398	0.946	0.923
**Hepatic rSO**_2_					
Withholding feeding group	55.9 (8.79)	57.68 (4.89)	57.29 (4.34)	57.35 (4.46)	56.92 (4.85)
Feeding group	56.56 (6.98)	56.83 (6.69)	57.28 (8.17)	56.46 (4.93)	56.05 (4.45)
*P*-value	0.825	0.685	0.995	0.604	0.612
**Renal rSO**_2_					
Withholding feeding group	53.77 (5.56)	58.06 (5.02)	57.29 (8.44)	57.52 (5.68)	58.62 (5.33)
Feeding group	55.97 (5.31)	57.77 (4.12)	56.22 (6.16)	54.38 (4.72)	53.2 (4.84)
*P*-value	0.313	0.874	0.723	0.145	0.012
**SCOR**					
Withholding feeding group	0.92 (0.18)	0.89 (0.12)	0.98 (0.26)	0.95 (0.19)	0.91 (0.10)
Feeding group	0.82 (0.10)	0.88 (0.08)	0.94 (0.14)	0.88 (0.12)	0.90 (0.08)
*P*-value	0.118	0.818	0.563	0.295	0.92

NIRS, near-infrared spectroscopy; rSO_2_, regional oxygen saturation; SCOR, splanchnic cerebral oxygenation ratio; SD, standard deviation.

Significant changes in the renal rSO_2_ and FOE (Table 3 and Figure 3) were observed between groups at 48 h after transfusion. An interaction between renal oxygenation and FOE with the feeding protocol techniques was noted. At 48 h after transfusion, the renal rSO_2_ and FOE of the withholding feeding group were significantly different from those of the feeding group; however, no significant difference was observed at other time points.

Differences in clinical outcomes were not observed (Table 4). One infant in the withholding feeding group developed gastrointestinal symptoms 2 h after transfusion and was diagnosed with NEC. No cases of death occurred. No difference in the FI incidence between groups was observed (21.4 vs. 7.7%; *P* = 0.331).

**Table 4:** Clinical outcomes.

	**Withholding feeding group**	**Feeding group**	***P*-value**
NEC, *N* (%)	0	0	
TA-NEC, *N* (%)	1 (3.6)	0	0.331
FI, *N* (%)	6 (21.4)	2 (7.7)	0.156
BPD, *N* (%)	14 (50)	13 (50)	1
ROP, *N* (%)	12 (42.9)	15 (57.7)	0.256

BPD, bronchopulmonary dysplasia; FI, feeding intolerance; NEC, necrotizing enterocolitis; TA-NEC, transfusion-associated necrotizing enterocolitis; ROP, retinopathy of prematurity.

No increase in IVH incidence was observed. During this study, the BPD incidence rate was 50% in both groups; however, the ROP incidence rates were 42.9% in the withholding feeding group and 57.7% in the feeding group, with no significant difference between groups. A subsequent investigation revealed no association between BPD and the total volume of the RBC transfusions (cc/kg) [*R*^2^ = 0.228; *P* = 0.218 (>0.05)]; however, a strong positive correlation was observed between ROP and the number of transfusions (*R*^2^ = 0.42; *P* = 0.021(<0.05)].

## Discussion

Recently, NIRS monitoring of rSO_2_ has been widely applied to manage critically ill infants in the NICU. Human cerebral circulation has a certain degree of autonomous regulation, and the cerebral rSO_2_ value is relatively stable. However, gastrointestinal rSO_2_ is easily affected by the infant's position, intestinal peristalsis, and feeding factors, and high variability has been observed among different individuals (29). Therefore, this study not only directly compared rSO_2_ but also measured intestinal and cerebral rSO_2_ simultaneously. Subsequently, the SCOR was calculated to eliminate factors that influence intestinal rSO_2_. Additionally, we recorded the SpO_2_ of the patients and calculated the local tissue oxygen uptake fraction (FTOE) to reflect oxygen use in tissues. By these measurements, more accurate comparisons between groups and individuals within a group were possible.

A blood transfusion is a common therapy administered in the NICU. Blood transfusions for premature infants can lead to TA-NEC, which is closely related to IVH, BPD, and ROP (30–32). Additionally, the pathogenesis of TA-NEC may be closely related to intestinal mucosal hypoxic-ischemic reperfusion injury, inflammatory cascade reactions, and intestinal microbiota imbalance caused by anemia (8, 10, 16, 33). Withholding feeding decreased the risk of TA-NEC (13). When feeding is continued during blood transfusions, gut oxygenation in premature infants shows a declining trend, suggesting hypoxia-reperfusion injury and a probable association with TA-NEC development (34). This study found no significant differences between the peri-transfusion rates of cerebral oxygenation, intestinal oxygenation, liver oxygenation, FTOE, and SCOR. Additionally, no significant variations in gastrointestinal and preterm complications The findings of this study are consistent with those of the study by Schindler et al. (15), who randomly divided preterm infants who received blood transfusion into three groups (withholding enteral feeds for 12 h group, continuing enteral feeds, and restricting the enteral feed volume to 120 cc/kg/day group). Schindler et al. (15) compared the intestinal to-brain oxygen ratio (SCOR) and FTOE values of those three groups transfusion 1 h before transfusion, immediately after transfusion, and 12 and 24 h transfusion. No significant differences in the average SCOR and FTOE were observed among those three groups at any of the aforementioned times, and no difference in the TA-NEC incidence was observed (15). The gestational age, birth weight, postnatal age at the time of transfusion, and hemoglobin levels of the infants before transfusion observed during the present study are consistent with those observed by Schindler et al. (15). However, the average enteral feed volume of the feeding group was lower than that observed during this study (66.31 ± 47.50 ml/kg). This may have occurred because our hospital does not have a breast milk bank. Most infants in our NICU lived far from the hospital. During hospitalization, most infants received mixed feedings and a conservative protocol for adding milk. The feed withholding time during this study was shorter than that; however, no differences in the incidence of TA-NEC and other symptoms of the feeding and withholding enteral feeding groups were observed. The intestinal transit times of premature infants are four-times greater than those of full-term infants; therefore, a 4-h fast may not have the desired effect of lowering NEC (35). In contrast to the studies by Schindler et al. and others (15, 36), we adopted a protocol involving an 8-h feeding withholding period for the withholding feeding group, from the start of the transfusion. This study indicated that withholding enteral feeding for shorter periods (<12 h) during and after the transfusion may be safe.

According to previous studies of TA-NEC, most patients received multiple RBC transfusions (37). For example, Mohankumar et al. (16) developed a neonatal murine model of TA-NEC and demonstrated that multiple RBC transfusions increased the risk of bowel injury. Therefore, we limited our study to infants who received one or more blood transfusions before the study. Most studies did not specify the frequency of blood transfusions received by the enrolled infants. Therefore, this study is more in line with TA-NEC high-risk factors.

During this study, we compared the changes in splanchnic oxygenation in infants in the same group at different time points before and after transfusion. Splanchnic oxygenation in infants in both groups showed an increasing trend immediately after the transfusion. However, the increase in intestinal oxygenation in the feeding group was significant. In contrast, no significant difference was observed in the withholding enteral feeding group. The same feeding protocol group experienced no significant changes in intestinal oxygenation at 8, 24, and 48 h after the transfusion. These results indicated that feeding premature infants during blood transfusions may temporarily increase intestinal oxygenation. However, no significant differences in the SCOR and FOE were observed at different time points before and after transfusions within the same group, and no significant difference between the different feeding strategy groups was observed. Under normal circumstances, enteral feeding promotes the release of substance P by the central nervous system, thus leading to intestinal mucosal vasodilation, increased intestinal blood flow, and oxygen delivery (38). Braski et al. reported that enteral feeding led to a decreased SCOR for infants with anemia and a hematocrit level <28%, which may lead to decreased intestinal perfusion and increased risks of FI and NEC (32). Blood transfusions can also lead to decreased postprandial intestinal oxygen utilization efficiency and splanchnic tissue oxygen levels (34, 39, 40). The difference between the results of this study and those of the study by Balegar et al. may be related to different feeding strategies and the storage time of infused RBCs (39). Balegar et al. reported the use of a feeding protocol with a syringe pump at a constant speed (120 ml/h); however, we adopted a feeding protocol involving gravity. Furthermore, in the study by Balegar et al., the median age of the transfused RBCs was 9 days; however, in our study, RBCs were used within 7 days of blood collection. The storage time of banked blood may affect the ability of RBCs to release oxygen (41). The hematocrit (HCT) level before transfusion during our study was 25% which was lower than that during Braski et al.'s (26.3%), Marin et al.'s study (26%) and Balegar et al.'s study (Hb 9.3 g/dl). Therefore, the level of anemia could not have led to the difference between our research results and that of previous studies. Marin et al. (34) compared the intestinal oxygenation of preterm infants who were fed and those of preterm infants who were not fed during RBC transfusion (all had a gestational age <33 weeks); however, they did not provide information regarding cerebral oxygenation and oxygen saturation. In the study by Marin et al. (34), decreased postprandial intestinal oxygenation mainly occurred in preterm infants with a postmenstrual age <33 weeks, and especially in those with a postmenstrual age <30 weeks. Conversely, in the present study, most preterm infants had a postmenstrual age ≥34 weeks. However, the median postmenstrual age of the infants in our study was very similar with that of in the study of Braski et al. and Balegar et al. Therefore, it was not yet certain that infants with lower postmenstrual age have greater risk for altered gut rSO_2_ during reperfusion phase following transfusion.

In this study, no significant changes were observed in the liver and brain oxygen levels before and after transfusion. However, the renal rSO_2_ levels significantly increased and the renal FTOE levels decreased in the withholding feeding group, but no significant change in renal oxygenation was observed in the feeding group. No significant differences in the renal function, renal tissue oxygenation, baseline hemoglobin and hematocrit levels, caffeine use, or diuretic use were observed between the two groups before blood transfusion. However, in the feeding group, a significant increase in intestinal oxygen content was observed immediately after blood transfusion. The increased renal tissue oxygenation in the withholding feeding group may have been caused by the differences in feeding strategies, leading to different blood flow distribution ratios between organs after blood transfusion. We did not perform routine ultrasound examinations of renal hemodynamics during this study; therefore, we cannot rule out the possibility that differences may exist in renal hemodynamics before blood transfusion in patients with different feeding strategies, leading to differences in renal oxygenation after blood transfusion.

Currently, the effects of blood transfusions on the kidneys are unclear, and conflicting results of the effects of different transfusion thresholds on renal blood flow before and after transfusion have been reported (42–44). Limited research of the effects of renal oxygen before and after transfusion has been performed; therefore, further research is required.

Prior studies have shown that infants who were fed formula are at higher risk for TA-NEC (37), but that breastfeeding can reduce the risk of NEC (45). However, during this study, few infants were exclusively breastfed and fortified with breast milk; the majority received breast milk and formula. However, the sample size was small, and group comparisons were insufficient.

The primary strength of this study is that we controlled for corresponding confounding factors. This study implemented a blind method for NIRS measurements, thus eliminating subjective bias and allowing more reliable results. Thirty minutes before and after the corresponding time points of the measurement, nurses checked the position of the probe and did not perform nursing procedures to ensure that the child was quiet and did not experience any changes in body position, thus avoiding the effects of the probe or body position changes on the child. The feeding group received a standardized feeding method after transfusion that involved gravity feeding for 5–10 min.

This study had some limitations. First, we examined the effects of various feeding methods on splanchnic tissue oxygenation during the peri-transfusion period, but we did not monitor the splanchnic tissue oxygenation changes during transfusion and splanchnic hemodynamics. Second, we did not continuously monitor rSO_2_. The NIR monitoring instrument used during our study was the EGOS-600B near-infrared blood oxygen monitoring instrument (Aiqin Medical Biomedical Electronics), which collected tissue rSO_2_ data every 2 s. However previous studies have indicated that measuring rSO_2_ within 10 min could provide accurate values when those values were stabilized (with fluctuations not exceeding 2%). Most of the research conducted with the use of this device adopted intermittent monitoring rather than continuous monitoring. Because of the lack of cameras and other software in our NICU, we were unable to ensure accurate recordings of infant movements and manage movement artifacts. Therefore, we chose several rSO_2_ measurement time points. We used clear monitoring time points for each patient in this study to ensure the scientific validity of our research data. Additionally, because of the small sample size, we could not completely rule out the possibility of a second type of error that may have affected findings and reduced the importance of some differences. Infants with hemodynamic instability are at high risk for TA-NEC; therefore, were excluded from this study. Another reason for the lack of difference in the TA-NEC incidence between groups may have been the relatively high hemoglobin levels of the included infants. Patel et al. (46) reported that hemoglobin levels <8 g/dl significantly increased the incidence of TA-NEC. An in-depth study with a larger sample size is required.

## Conclusions

The results of this study showed that splanchnic oxygenation in very preterm infants during the transfusion phase was unaffected by their feeding status. Feeding during RBC transfusion did not increase the incidences of intestinal ischemia after transfusion and TA-NEC in preterm infants compared with those of infants whose feedings were withheld during blood transfusions. Future studies should include multicenter trials with larger sample sizes to assess the long-term effects of different feeding strategies during RBC transfusions for preterm infants. Additionally, an investigation of the effects of the feeding volume, composition, RBC storage time, and hemodynamic monitoring on tissue oxygenation and transfusion-related complications would provide valuable insights regarding the optimization of clinical management protocols for this vulnerable population.

## Data availability statement

The original contributions presented in the study are included in the article/supplementary material, further inquiries can be directed to the corresponding authors.

## Ethics statement

The studies involving humans were approved by the medical Ethics Committee of West China Second University Hospital, Sichuan University. The studies were conducted in accordance with the local legislation and institutional requirements. Written informed consent for participation in this study was provided by the participants' legal guardians/next of kin.

## Author contributions

JH: Writing – original draft, Conceptualization, Formal analysis, Investigation. XS: Supervision, Conceptualization, Writing – original draft. XX: Data curation, Software, Writing – original draft. HL: Formal analysis, Methodology, Writing – original draft. JT: Validation, Project administration, Writing – review & editing. TX: Resources, Visualization, Writing – review & editing. JZ: Supervision, Writing – review & editing. JS: Resources, Writing – review & editing.

## References

[1] Keir AK Yang J Harrison A Pelausa E Shah PS Network CN. Temporal changes in blood product usage in preterm neonates born at less than 30 weeks' gestation in Canada. Transfusion. (2015) 55:1340–6. 10.1111/trf.12998 25652740

[2] Schat TE Heida FH Schurink M van der Laan ME Hulzebos CV Bos AF. The relation between splanchnic ischaemia and intestinal damage in necrotising enterocolitis. Arch Dis Child Fetal Neonatal Ed. (2016) 101:F533–9. 10.1136/archdischild-2015-309838 27048432

[3] Keir A Pal S Trivella M Lieberman L Callum J Shehata N. Adverse effects of red blood cell transfusions in neonates: a systematic review and meta-analysis. Transfusion. (2016) 56:2773–80. 10.1111/trf.13785 27600435

[4] Stritzke AI Smyth J Synnes A Lee SK Shah PS. Transfusion-associated necrotising enterocolitis in neonates. Arch Dis Child Fetal Neonatal Ed. (2013) 98:F10–4. 10.1136/fetalneonatal-2011-301282 22447991

[5] McGrady GA Rettig PJ Istre GR Jason JM Holman RC Evatt BL. An outbreak of necrotizing enterocolitis. Association with transfusions of packed red blood cells. Am J Epidemiol. (1987) 126:1165–72. 10.1093/oxfordjournals.aje.a114754 3687923

[6] Blau J Calo JM Dozor D Sutton M Alpan G La Gamma EF. Transfusion-related acute gut injury: necrotizing enterocolitis in very low birth weight neonates after packed red blood cell transfusion. J Pediatr. (2011) 158:403–9. 10.1016/j.jpeds.2010.09.015 21067771

[7] Mally P Golombek SG Mishra R Nigam S Mohandas K Depalhma H. Association of necrotizing enterocolitis with elective packed red blood cell transfusions in stable, growing, premature neonates. Am J Perinatol. (2006) 23:451–8. 10.1055/s-2006-951300 17009195

[8] Marin T Strickland OL. Transfusion-related necrotizing enterocolitis: a conceptual framework. Adv Neonatal Care. (2013) 13:166–74. 10.1097/ANC.0b013e318285f901 23722487

[9] Bailey SM Hendricks-Muñoz KD Mally PV. Variability in splanchnic tissue oxygenation during preterm red blood cell transfusion given for symptomatic anaemia may reveal a potential mechanism of transfusion-related acute gut injury. Blood Transfus. (2015) 13:429–34. 10.2450/2015.0212-14 25761320 PMC4614295

[10] Rose AT Saroha V Patel RM. Transfusion-related gut injury and necrotizing enterocolitis. Clin Perinatol. (2020) 47:399–412. 10.1016/j.clp.2020.02.002 32439119 PMC7245583

[11] Martini S Spada C Aceti A Rucci P Gibertoni D Battistini B. Red blood cell transfusions alter splanchnic oxygenation response to enteral feeding in preterm infants: an observational pilot study. Transfusion. (2020) 60:1669–75. 10.1111/trf.15821 32358809

[12] Morini F Bagolan P. Transfusion-related necrotizing enterocolitis. J Pediatr. (2011) 159:701–3. 10.1016/j.jpeds.2011.05.047 21784439

[13] Jasani B Rao S Patole S. Withholding feeds and transfusion-associated necrotizing enterocolitis in preterm infants: a systematic review. Adv Nutr. (2017) 8:764–9. 10.3945/an.117.015818 28916576 PMC5593105

[14] Yeo KT Kong JY Sasi A Tan K Lai NM Schindler T. Stopping enteral feeds for prevention of transfusion-associated necrotising enterocolitis in preterm infants. Cochrane Database Syst Rev. (2019) 10.1002/14651858.CD012888.pub2 31684689 PMC6815687

[15] Schindler T Yeo KT Bolisetty S Michalowski J Tan AHK Lui K. FEEding DURing red cell transfusion (FEEDUR RCT): a multi-arm randomised controlled trial. BMC Pediatr. (2020) 20:346. 10.1186/s12887-020-02233-3 32664953 PMC7359615

[16] MohanKumar K Namachivayam K Song T Jake Cha B Slate A Hendrickson JE. A murine neonatal model of necrotizing enterocolitis caused by anemia and red blood cell transfusions. Nat Commun. (2019) 10:3494. 10.1038/s41467-019-11199-5 31375667 PMC6677753

[17] Marin T Moore JE. Mesenteric oxygenation changes associated with necrotizing enterocolitis and pneumoperitoneum after multiple blood transfusions: a case report. Adv Neonatal Care. (2018) 18:121–7. 10.1097/ANC.0000000000000461 29300196

[18] New HV Berryman J Bolton-Maggs PH Cantwell C Chalmers EA Davies T. Guidelines on transfusion for fetuses, neonates and older children. Br J Haematol. (2016) 175:784–828. 10.1111/bjh.14233 27861734

[19] Chock VY Smith E Tan S Ball MB Das A Hintz SR. Early brain and abdominal oxygenation in extremely low birth weight infants. Pediatr Res. (2022) 92:1034–41. 10.1038/s41390-022-02082-z 35513716 PMC9588487

[20] Amigoni A Mozzo E Brugnaro L Tiberio I Pittarello D Stellin G. Four-side near-infrared spectroscopy measured in a paediatric population during surgery for congenital heart disease. Interact Cardiovasc Thorac Surg. (2011) 12:707–12. 10.1510/icvts.2010.253328 21335618

[21] Navikiene J Virsilas E Vankeviciene R Liubsys A Jankauskiene A. Brain and renal oxygenation measured by NIRS related to patent ductus arteriosus in preterm infants: a prospective observational study. BMC Pediatr. (2021) 21:559. 10.1186/s12887-021-03036-w 34886825 PMC8656008

[22] Li R Ye X Li G Cao X Zou Y Yao S. Effects of different body positions and head elevation angles on regional cerebral oxygen saturation in premature infants of China. J Pediatr Nurs. (2020) 55:1–5. 10.1016/j.pedn.2020.05.014 32570090

[23] Yang X Lei X Zhang L Zhang L Dong W. The application of near-infrared spectroscopy in oxygen therapy for premature infants. J Matern Fetal Neonatal Med. (2020) 33:283–8. 10.1080/14767058.2018.1489535 29898632

[24] van Bel F Lemmers P Naulaers G. Monitoring neonatal regional cerebral oxygen saturation in clinical practice: value and pitfalls. Neonatology. (2008) 94:237–44. 10.1159/000151642 18784420

[25] Fanaro S. Feeding intolerance in the preterm infant. Early Hum Dev. (2013) 89(Suppl 2):S13–20. 10.1016/j.earlhumdev.2013.07.013 23962482

[26] Paul DA Mackley A Novitsky A Zhao Y Brooks A Locke RG. Increased odds of necrotizing enterocolitis after transfusion of red blood cells in premature infants. Pediatrics. (2011) 127:635–41. 10.1542/peds.2010-3178 21402638

[27] Sandal G Oguz SS Erdeve O Akar M Uras N Dilmen U. Assessment of red blood cell transfusion and transfusion duration on cerebral and mesenteric oxygenation using near-infrared spectroscopy in preterm infants with symptomatic anemia. Transfusion. (2014) 54:1100–5. 10.1111/trf.12359 23901886

[28] Dani C Pratesi S Fontanelli G Barp J Bertini G. Blood transfusions increase cerebral, splanchnic, and renal oxygenation in anemic preterm infants. Transfusion. (2010) 50:1220–6. 10.1111/j.1537-2995.2009.02575.x 20113454

[29] Marin T Moore J. Understanding near-infrared spectroscopy: an update. Crit Care Nurs Clin North Am. (2024) 36:41–50. 10.1016/j.cnc.2023.08.001 38296375

[30] Banerjee J Leung TS Aladangady N. Cerebral blood flow and oximetry response to blood transfusion in relation to chronological age in preterm infants. Early Hum Dev. (2016) 97:1–8. 10.1016/j.earlhumdev.2015.10.017 26619762

[31] Whitehead HV Vesoulis ZA Maheshwari A Rao R. Mathur AM. Anemia of prematurity and cerebral near-infrared spectroscopy: should transfusion thresholds in preterm infants be revised? J Perinatol. (2018) 38:1022–9. 10.1038/s41372-018-0120-0 29740185 PMC6136959

[32] Braski K Weaver-Lewis K Loertscher M Ding Q Sheng X Baserga M. Splanchnic-cerebral oxygenation ratio decreases during enteral feedings in anemic preterm infants: observations under near-infrared spectroscopy. Neonatology. (2018) 113:75–80. 10.1159/000481396 29131125 PMC5734057

[33] Arthur CM Nalbant D Feldman HA Saeedi BJ Matthews J Robinson BS. Anemia induces gut inflammation and injury in an animal model of preterm infants. Transfusion. (2019) 59:1233–45. 10.1111/trf.15254 30897226 PMC6525338

[34] Marin T Josephson CD Kosmetatos N Higgins M Moore JE. Feeding preterm infants during red blood cell transfusion is associated with a decline in postprandial mesenteric oxygenation. J Pediatr. (2014) 165:464–71. 10.1016/j.jpeds.2014.05.009 24948351

[35] Killion E. Feeding Practices and effects on transfusion-associated necrotizing enterocolitis in premature neonates. Adv Neonatal Care. (2021) 21:356–64. 10.1097/ANC.0000000000000872 33938478

[36] El-Dib M Narang S Lee E Massaro AN Aly H. Red blood cell transfusion, feeding and necrotizing enterocolitis in preterm infants. J Perinatol. (2011) 31:183–7. 10.1038/jp.2010.157 21252964

[37] Christensen RD Lambert DK Henry E Wiedmeier SE Snow GL Baer VL. Is “transfusion-associated necrotizing enterocolitis” an authentic pathogenic entity? Transfusion. (2010) 50:1106–12. 10.1111/j.1537-2995.2009.02542.x 20051059

[38] Reber KM Nankervis CA Nowicki PT. Newborn intestinal circulation. Physiol Pathophysiol Clin Perinatol. (2002) 29:23–39. 10.1016/S0095-5108(03)00063-0 11917738

[39] Balegar V KK Jayawardhana M Martin AJ de Chazal P Nanan RKH. Association of bolus feeding with splanchnic and cerebral oxygen utilization efficiency among premature infants with anemia and after blood transfusion. JAMA Netw Open. (2020) 3:e200149. 10.1001/jamanetworkopen.2020.0149 32108891 PMC7049081

[40] Wan-Huen P Bateman D Shapiro DM Parravicini E. Packed red blood cell transfusion is an independent risk factor for necrotizing enterocolitis in premature infants. J Perinatol. (2013) 33:786–90. 10.1038/jp.2013.60 23702619

[41] Li Y Xiong Y Wang R Tang F Wang X. Blood banking-induced alteration of red blood cell oxygen release ability. Blood Transfus. (2016) 14:238–44. 10.2450/2015.0055-15 26674824 PMC4918555

[42] Fitzgibbons SC Ching Y Yu D Carpenter J Kenny M Weldon C. Mortality of necrotizing enterocolitis expressed by birth weight categories. J Pediatr Surg. (2009) 44:1072–6. 10.1016/j.jpedsurg.2009.02.013 19524719

[43] Quante M Pulzer F Bläser A Gebauer C Kluge J Robel-Tillig E. Effects of anaemia on haemodynamic and clinical parameters in apparently stable preterm infants. Blood Transfus. (2013) 11:227–32. 10.2450/2012.0171-11 22871817 PMC3626473

[44] Jani P Lowe K Hinder M Galea C D'Çruz D Badawi N. Changes to hepatic tissue oxygenation, abdominal perfusion and its association with enteral feeding with liberal transfusion threshold in anaemic preterm infants: a prospective cohort study. Vox Sang. (2020) 115:712–21. 10.1111/vox.12938 32424842

[45] Sullivan S Schanler RJ Kim JH Patel AL Trawöger R Kiechl-Kohlendorfer U. An exclusively human milk-based diet is associated with a lower rate of necrotizing enterocolitis than a diet of human milk and bovine milk-based products. J Pediatr. (2010) 156:562–7. 10.1016/j.jpeds.2009.10.040 20036378

[46] Patel RM Knezevic A Shenvi N Hinkes M Keene S Roback JD. Association of red blood cell transfusion, anemia, and necrotizing enterocolitis in very low-birth-weight infants. JAMA. (2016) 315:889–97. 10.1001/jama.2016.1204 26934258 PMC4805423

